# Mirtazapine: A One-Drug Intervention for the Physical and Psychological Symptoms of End-Stage Renal Disease (ESRD)

**DOI:** 10.7759/cureus.71439

**Published:** 2024-10-14

**Authors:** Onyinyechi V Evoh, Totini Chatterjee, Sean O'Mahony, Mukaila Raji

**Affiliations:** 1 Department of Internal Medicine, Division of Palliative Care, University of Texas Medical Branch at Galveston, Galveston, USA; 2 Department of Internal Medicine, Division of Geriatrics, University of Texas Medical Branch at Galveston, Galveston, USA; 3 Department of Preventive Medicine and Population Health, University of Texas Medical Branch at Galveston, Galveston, USA

**Keywords:** end-stage renal disease (esrd), mirtazapine, palliative care, polypharmacy management, symptom management

## Abstract

Patients with end-stage renal disease (ESRD), especially those on dialysis, experience myriad medical and psychological symptoms that impact theirquality of life. These symptoms range from nausea, emesis, and insomnia to pruritus, anxiety, depression, and loss of appetite. These symptoms often require multiple medications (e.g., anticholinergics, antihistamines, tricyclics, benzodiazepines, and Z-drugs), which can lead to polypharmacy, medication non-adherence, and potentially harmful drug-drug interactions, especially in older ESRD patients, a population with an age-related decline in drug metabolism, multimorbidity, and polypharmacy. The current perspective article will discuss evidence from extant literature supporting mirtazapine (a noradrenergic antagonist and selective serotonin antagonist, NASSA) as a potentially useful one-stop pharmacologic agent to alleviate multiple symptoms that ESRD patients face. A one-stop strategy has the potential to decrease polypharmacy, reduce adverse drug-drug and drug-disease interactions, reduce healthcare costs, and improve the quality of life in ESRD patients. Mirtazapine use in an ESRD setting merits a large randomized pragmatic double-blinded controlled trial to evaluate its efficacy as a potential pharmacotherapeutic agent for the management of multiple disabling gastrointestinal and other neuropsychological symptoms in older adults living with ESRD.

## Introduction and background

More than 500,000 people in the United States currently live with end-stage renal disease (ESRD), which occurs when a person's kidney function has permanently ceased [1]. Patients who have ESRD and desire life-prolonging interventions will require either dialysis or kidney transplantation. The United Network for Organ Sharing reported a record-breaking number of transplants in 2023 at 27,000 [2], but this number still leaves most patients with ESRD dependent on either peritoneal or hemodialysis. Dialysis can extend the duration of life, but patients undergoing dialysis still face many medical and psychological symptoms that can impact their quality of life. These symptoms range from gastrointestinal disorders (e.g., nausea, dyspepsia, and emesis), sleep disorders, skin discomfort (e.g., pruritus), pain, depression, anxiety, and loss of appetite [3-6]. These symptoms are common and debilitating and adversely affect the overall quality of life for ESRD patients. Many studies and medications have attempted to address these symptoms and propose solutions to the suffering that ESRD patients face. However, patients often require multiple medications to control their symptoms, which can lead to polypharmacy, medication non-adherence, and harmful drug-drug interactions in a population that already has a reduced metabolism of medications. Instead of relying on multiple medications for symptom management in ESRD patients, we would instead like to propose mirtazapine as a one-drug regimen [7-10].

Mirtazapine is a tetracyclic/atypical antidepressant that acts as a central alpha-2 antagonist, leading to increased norepinephrine and serotonin release. It is also an antagonist at 5-HT2 and 5-HT3 serotonin and H1 histamine receptors and has moderate antagonist action at peripheral alpha-1 and muscarinic receptors [3]. It was initially approved for the treatment of major depressive disorder (MDD) in the Netherlands in 1994 and received FDA approval for the treatment of MDD in 1997 [3]. It has been shown to alleviate symptoms of depression and has also been shown to improve several other symptoms, including insomnia, nausea, and pain. Its effects may manifest within just two weeks [11]. The current perspective article will discuss the evidence from a review of extant literature supporting mirtazapine as a potentially useful one-stop pharmacologic agent to alleviate many of the symptoms that ESRD patients face. A one-stop strategy has the potential to decrease polypharmacy, reduce adverse drug-drug and drug-disease interaction events, reduce healthcare costs, and improve quality of life [7-10].

## Review

Pruritus

One of the most common symptoms faced by dialysis patients is pruritus [12]. Pruritus can be a bothersome and even debilitating symptom experienced by many patients with renal disease. Uremia is thought to be a major cause of pruritus experienced by dialysis patients, likely from a combination of increased systemic inflammation, abnormal serum parathyroid hormone, calcium and phosphorus levels, and an imbalance in opioid receptors and the neuropathic process [13]. However, pruritus is also believed to be mediated via histamine through H1 receptors and serotonin at 5-HT2 and 5-HT3 peripheral receptors [14]. Mirtazapine may prove to be a useful tool in alleviating pruritus in patients with renal disease due to its antagonistic action at these receptors. Mirtazapine is already used clinically to treat pruritus in many conditions, including liver failure, uremic cholestasis, psoriasis, atopic dermatitis, primary and metastatic cancers, lymphoma, and leukemia [14]. In fact, a recent study even showed that when comparing mirtazapine to gabapentin, both the patient's perception of itching at the end of the trial period and the perception of change were greatly diminished [15]. In addition, case reports specifically detail its effectiveness at alleviating pruritus in patients with ESRD, most often at the 15 mg dose [14]. These findings would support that mirtazapine could be a beneficial medication to alleviate pruritus in ESRD patients [7-10,16].

Insomnia

Similar to pruritus, the exact pathogenesis of insomnia in patients with renal disease remains unknown. Some of the proposed mechanisms include chronic inflammation, elevated plasma levels of orexin (a neuropeptide that promotes wakefulness), and sleep disruption related to dialysis treatment itself [17]. Mirtazapine has a high affinity to histamine H1, 5-HT2A, and 5-HT2C receptors at doses of 15 mg and lower, which partially mediates sedation and promotes sleep [18]. Mirtazapine can, therefore, be considered a medication to improve sleep, especially as an astonishingly considerable number of dialysis patients suffer from insomnia [17]. The beneficial effects on sleep have also been demonstrated in other populations, including the older adult and cancer populations [19,20]. In addition, a two-week parallel trial of 15 mg versus 30 mg daily mirtazapine in 130 depressed patients with insomnia demonstrated improvement in sleep quality and quantity, ease of getting to sleep, and daytime alertness with both doses [21]. Other studies have also shown an increase in total sleep time and a reduction in nighttime awakenings with the use of mirtazapine [19]. It can, therefore, be inferred that ESRD patients would benefit from mirtazapine for better sleep quality and quantity. Perhaps most importantly, given the number of medications most dialysis patients are prescribed, mirtazapine may be a better sleep aid when compared to other sleep aids due to its relatively fewer drug-drug interactions and a more favorable safety profile [7-10].

Pain

Pain is a common issue among ESRD patients, and multiple studies and case reports have attempted to explain and quantify the prevalence of chronic pain among dialysis patients. Many agents have been proposed to alleviate this common symptom, including opiates, gabapentinoids, and antidepressants [22]. Unfortunately, there is a scarcity of studies and case reports on mirtazapine's utility for pain, specifically in the ESRD population. However, given its efficacy in other chronic pain syndromes, it can be inferred that mirtazapine would also benefit ESRD patients who experience chronic pain because of their renal disease and other chronic pain syndromes. Several case reports have been published that help to support the utility of mirtazapine for patients with chronic pain, especially in patients whose pain is concurrent with depression. A report from the Journal of Pain and Symptom Management described a patient with chronic back pain due to an injury from a traumatic fall. The patient subsequently developed depression, and neither their pain nor mood showed improvement with multiple medications. The patient's mood and pain finally improved with the addition of 15 mg of mirtazapine [23]. Another study queries 594 patients with chronic pain syndrome and depression who were enrolled in a study and treated with mirtazapine. There was a statistically significant reduction of pain from baseline [24]. Patients in this study with neuropathic pain especially responded better to mirtazapine. These studies demonstrate that mirtazapine may be a useful tool for alleviating the pain and discomfort that ESRD patients face.

Mood disorders/depression

Mirtazapine has been approved by the FDA for the treatment of depression since 1997, and its use in practice for the treatment of depression is widely accepted. Depression is prevalent in ESRD patients and significantly diminishes their quality of life. A meta-analysis and systematic review published by Palmer et al. found that the prevalence of depression in ESRD patients was 39.3% when evaluated via screening questionnaires [25]. Another study found that patients undergoing dialysis in urban settings had a depression prevalence of 20%-30% [26]. Mirtazapine has been shown to improve depressive symptoms by inhibiting presynaptic alpha-2 adrenergic receptors, leading to increased release of serotonin and norepinephrine [27]. A medication such as mirtazapine that not only addresses the symptoms of depression but also aims to alleviate the other symptoms outlined above should be considered in patients with ESRD to improve their mood and overall quality of life.

Fatigue

Patients undergoing hemodialysis are constantly in a catabolic state. This state is caused by alterations in protein metabolism, increases in energy expenditure, and changes in protein needs due to inflammation [28]. This can ultimately lead to a patient feeling fatigued. As described above, depression and sleep disorders can contribute to a reduced quality of life and may also contribute to the overall feeling of fatigue expressed by dialysis patients. However, the catabolic state that is experienced by dialysis patients may also contribute to feelings of fatigue. Mirtazapine in and of itself certainly cannot reverse the catabolic state experienced by dialysis patients. However, its contribution to alleviating sleep disturbances and mood disorders may lead to improvements in fatigue levels experienced by dialysis patients. Furthermore, the improvement in sleep experienced with the addition of mirtazapine may help to minimize some of the harm created by the constant catabolic state of a dialysis patient. Lamon et al. showed that sleep deprivation can induce anabolic resistance and promote a catabolic state [29]. Improving sleep quality with mirtazapine may, therefore, alleviate some of the harmful effects of the pro-catabolic state experienced by dialysis patients and improve their overall fatigue levels.

Nausea/vomiting

Nausea and vomiting are highly prevalent symptoms in ESRD patients and may be experienced by up to 26% of this population [5,30]. Several factors can contribute to nausea in the ESRD population, including uremia, electrolyte imbalances, gastroparesis, fluid overload leading to mucosal edema, hypotension during hemodialysis sessions, and anxiety [31,32]. Many medications have been proposed to alleviate nausea in the ESRD population, including metoclopramide, haloperidol, and ondansetron, and mirtazapine may be an effective medication to add to this list. Mirtazapine has been shown to be an effective anti-emetic due to its antagonism at the 5-HT3 receptor [33]. Studies and case reports have demonstrated its effectiveness at treating nausea in patients after gastric bypass [34], with nausea due to chemotherapy [35] and nausea due to gastroparesis [36]. Unfortunately, case reports and studies that support mirtazapine's efficacy, specifically in the ESRD population, are scarce. However, as mirtazapine has been shown to be an effective anti-emetic in many other patient populations, we propose that mirtazapine has the potential to address the symptoms of nausea and vomiting in the ESRD population as well.

Anorexia/poor appetite, weight loss, and dysgeusia

Poor appetite and weight loss are common in ESRD patients, and the etiology of these symptoms is multifactorial. Sustained systemic inflammation, metabolic acidosis, uremic toxin accumulation, renal cachexia syndrome, and protein energy wasting have all been proposed as causes of anorexia and weight loss in the ESRD population [37-39]. Food aversion, early satiety, and changes in taste and smell have also been reported by ESRD patients with poor appetites [40]. Many studies have described mirtazapine's effects on appetite and weight gain, with some studies supporting its efficacy and other studies unconvinced of its efficacy. In a study published by JAMA Oncology, patients who took mirtazapine did not see improvements in appetite but did have an increase in energy intake (mainly fat intake) compared to the placebo group [41]. Another study attempted to quantify both megestrol and mirtazapine's effects on weight and appetite in addition to other variables in patients with cancer cachexia. Weight improved in 38% of the patients, and appetite improved in 56% of the patients taking mirtazapine [42]. Another study specifically evaluated weight gain in ESRD patients and found that dry weight was improved with the addition of mirtazapine [43].

Poor appetite due to changes in taste should also be taken into account when considering the utility of mirtazapine. Mirtazapine may also lead to the resolution of taste disturbances in some patients, specifically those in which taste disturbances result from other medications. This, in turn, can lead to an improvement in appetite. In a case report of an 84-year-old patient taking levofloxacin, her taste disturbances resolved one week after the initiation of mirtazapine, which improved her appetite [44]. Therefore, it would be reasonable to consider mirtazapine in the ESRD population with poor appetite, particularly if their other medications are contributing to dysgeusia.

Clinical implications

Multiple medications, such as anticholinergics and tricyclics, can help with treating the multiple gastrointestinal and neuropsychological symptoms of ESRD, but mirtazapine can serve as a one-stop strategy in managing these conditions, potentially leading to less medication burden, reduced polypharmacy, fewer adverse drug effects, and lower costs. In summary, we advocate for clinicians to consider the use of mirtazapine as a one-stop medication for ESRD-associated symptoms of insomnia, anxiety, poor appetite, nausea, emesis, pruritus, and other common ESRD-related symptoms. In older ESRD patients in particular, mirtazapine is of particular importance because it can potentially reduce adverse drug-drug interactions in a population with a high prevalence of multiple co-occurring chronic diseases that require multiple medications. We propose a large, randomized, pragmatic, double-blinded controlled trial of mirtazapine for the management of multiple disabling gastrointestinal and neuropsychological symptoms in older adults living with ESRD.

## Conclusions

Patients with ESRD face myriad distressing symptoms, which become more frequent as patients transition to dialysis. The impact of these symptom clusters, especially the gastrointestinal discomfort symptom cluster, sleep disorder symptom cluster, skin discomfort symptom cluster, and mood symptom cluster, is heightened with increasing age, reflecting the intersectionality of age-related physiological changes, multimorbidity, and polypharmacy. The current perspective article highlighted clinically relevant studies mostly from non-renal conditions (e.g., cancer) to illustrate mechanisms by which mirtazapine could palliate gastrointestinal disorders (e.g., nausea, dyspepsia, and emesis), sleep disorders, skin discomfort (e.g., pruritus), pain, depression, anxiety, and loss of appetite that lower the quality of life of patients living with ESRD. Further support for mirtazapine's efficacy in these highlighted cases should be pursued with a large, randomized, pragmatic, double-blinded controlled trial of mirtazapine for the management of multiple disabling gastrointestinal and neuropsychological symptoms in older adults living with ESRD.
